# A Rare Case of Choroid Plexus Papilloma of the Third Ventricle in an Adult

**DOI:** 10.7759/cureus.9582

**Published:** 2020-08-06

**Authors:** Mohammed Hamza Shah, Mohamed Abdelhady, Ahmed Own, Ahmed Elsotouhy

**Affiliations:** 1 Body Imaging, Radiology, Hamad Medical Corporation, Doha, QAT; 2 Neuroradiology Section, Neuroscience Institute, Hamad Medical Corporation, Doha, QAT

**Keywords:** choroid plexus papilloma, choroid plexus tumor, colloid cyst, third ventricle neoplasm, foramen of monro

## Abstract

Colloid cysts are the commonest masses of the third ventricle. Third ventricle neoplasms are uncommon. They include tumors arising from the choroid plexus (papillomas, carcinomas), tumors arising from other than the choroid plexus (ependymomas, meningiomas), metastases, and lymphoma. Choroid plexus tumors usually occur in the lateral ventricle in children and fourth ventricle in adults, and often present with hydrocephalus. We herein describe the extremely rare occurrence of third ventricle choroid plexus papilloma in a 35-year-old man who presented to the emergency department with a long history of intermittent headaches, occasionally associated with photophobia. CT and MR imaging revealed a lobulated ovoid lesion in the third ventricle with minimal extension into the right lateral ventricle through the foramen of Monro, causing mild ventricular dilatation. Surgical resection was performed and histopathology revealed choroid plexus papilloma.

## Introduction

The choroid plexus within the ventricles is responsible for producing cerebrospinal fluid (CSF). It consists of epithelial cells, capillaries, and connective tissue [1]. Choroid plexus tumors (CPTs) account for less than 1% of adult brain tumors and less than 4% of pediatric brain tumors [2,3]. They often present with hydrocephalus, which could be due to the overproduction of CSF and/or due to the obstruction of CSF flow. On the basis of histology, they are classified into choroid plexus papilloma (CPP, WHO grade I), atypical CPP (WHO grade II), and choroid plexus carcino­ma (CPC, WHO grade III). CPPs are far more common than CPC; and overall, CPTs are more commonly encountered in children than in adults. They are usually located in the lateral ventricle in children and in the fourth ventricle in adults; only about 5% occur in the third ventricle [2,3].

Colloid cysts are the most common masses of the third ventricle, typically located at the anterior superior aspect of the third ventricle at the foramen of Monro. Less common masses of the third ventricle include neoplasms arising from the choroid plexus (CPTs including CPP, atypical CPP, CPC), neoplasms arising from other than the choroid plexus (such as ependymoma, meningioma, craniopharyngioma, chordoid glioma), metastases, lymphoma, vascular lesions, infectious lesions (e.g. tuberculosis) and congenital intraventricular cysts (such as arachnoid, ependymal and neuroepithelial cysts) [4]. Tumors of the third ventricle are uncommon, accounting for less than 1% of brain tumors [5,6]. Preoperative differentiation of neoplasms of the third ventricle based on imaging can be challenging due to overlap in imaging features. Third ventricle CPTs are extremely rare, more so in adults. We present a surgically proven case of CPP of the third ventricle in a 35-year-old man.

## Case presentation

A 35-year-old male patient presented to the emergency department complaining of recurrent frontal headaches for one year. The headache was usually right-sided, associated with right periorbital pain and occasionally photophobia. He had no history of trauma, vomiting, or fever. On examination, he was afebrile, alert, and oriented, and had no weakness or numbness. 

**Figure 1:**
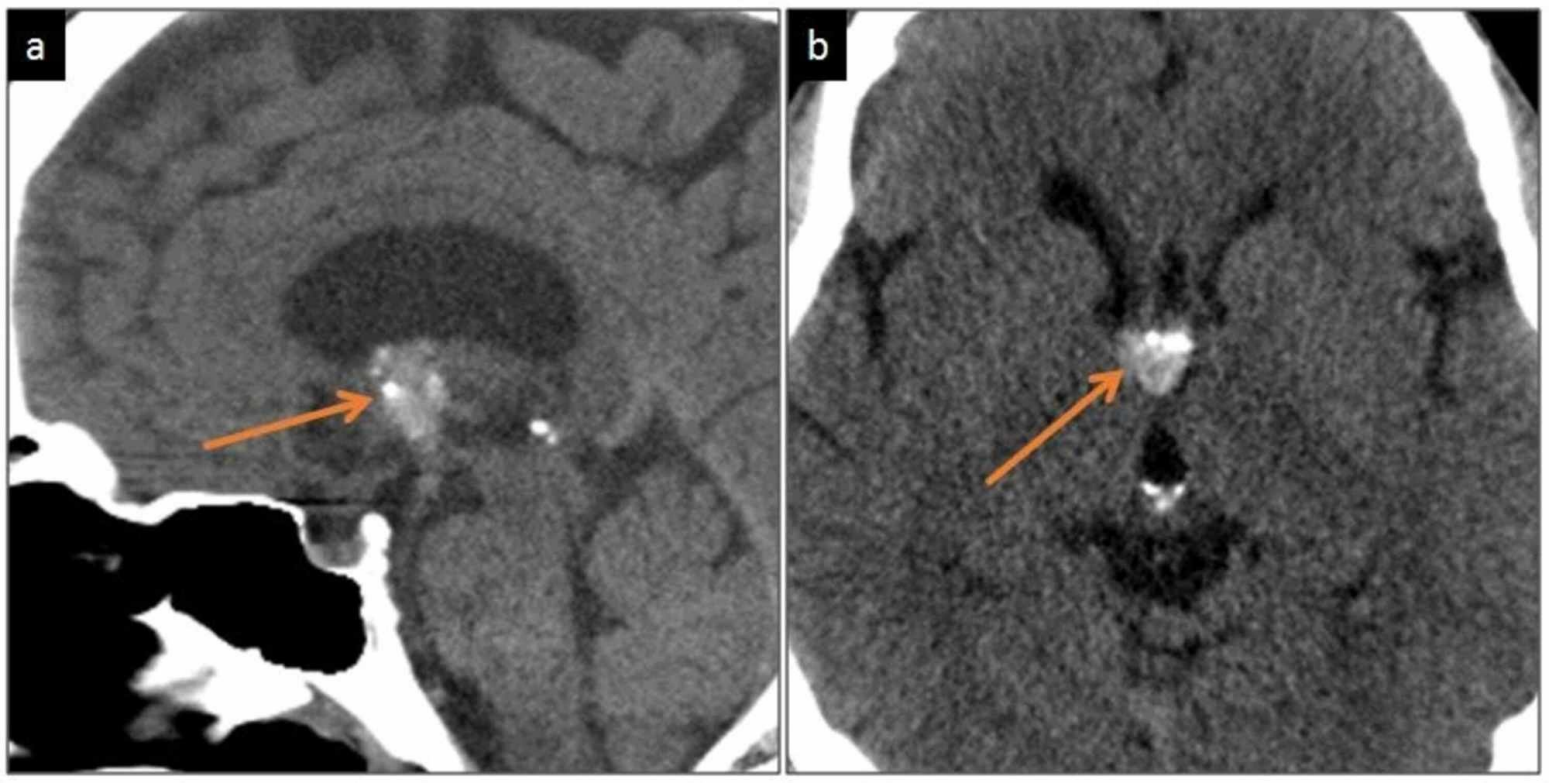
Unenhanced CT brain Sagittal (a) and axial (b) images demonstrate a hyperdense lesion (orange arrows) at the anterior third ventricle, extending to the foramen of Monro and protruding into the anterior horn of right lateral ventricle. It contains tiny calcifications. The right lateral ventricle is mildly dilated

An unenhanced CT scan of the head was performed, which revealed a lobulated ovoid well-defined hyperdense lesion at the anterior aspect of the third ventricle, extending into the foramen of Monro and slightly protruding into the anterior horn of right lateral ventricle (Figure 1). It measured 16 mm in maximal dimension, had a CT density of about 65 HU, and contained tiny specks of calcification. The anterior horn and body of the right lateral ventricle were mildly dilated compared to the left side. The possibility of a colloid cyst was raised and further evaluation by MRI was recommended. 

**Figure 2:**
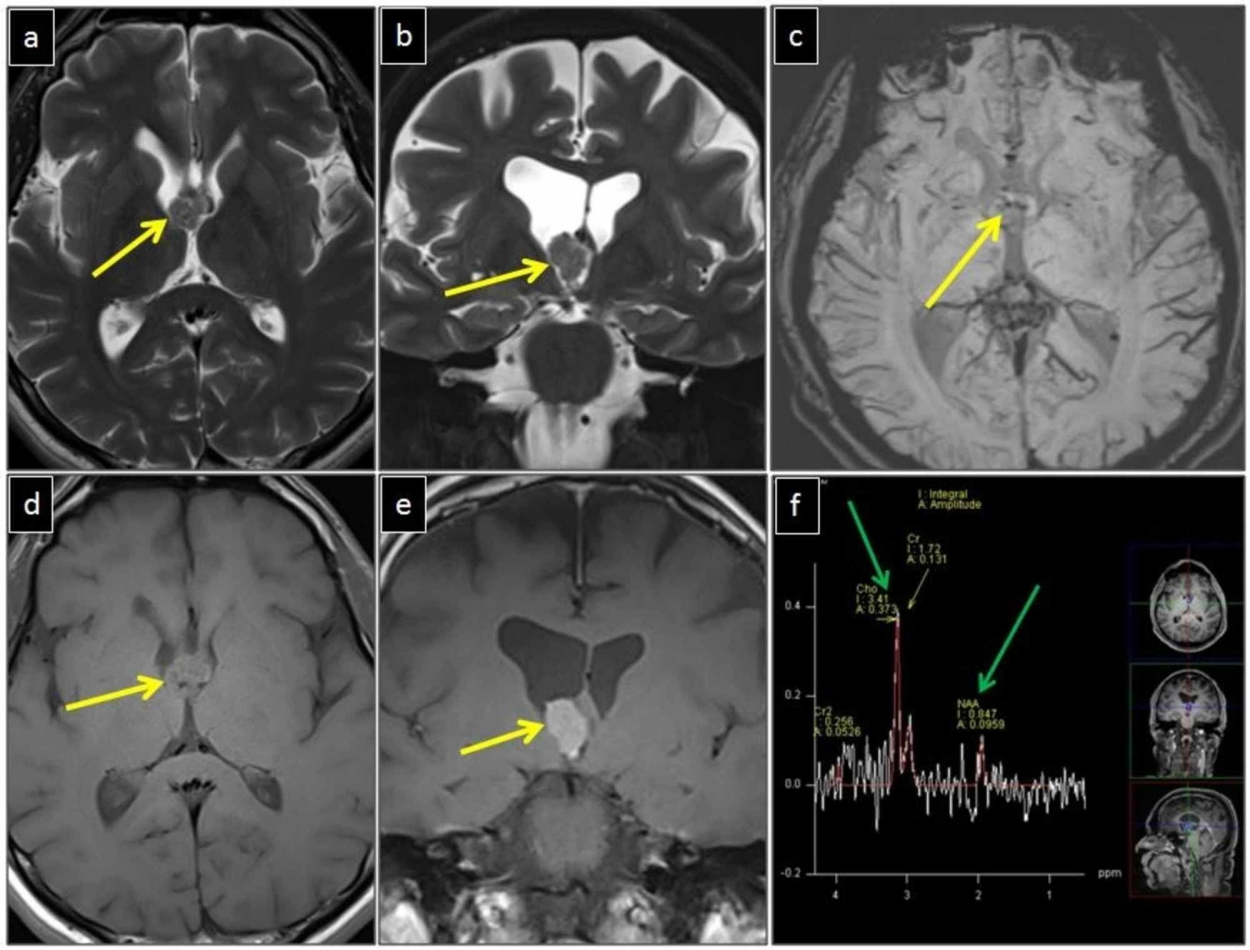
MRI brain The lesion (yellow arrows in a, b, d, e) in the anterior third ventricle has a lobulated outline. It is of intermediate signal on axial and coronal T2WI (a, b) and intermediate signal on axial T1WI (d). It shows intense homogeneous enhancement on coronal post-contrast images (e).  A few internal dark foci of blooming present on SWI (yellow arrow in c). MRS shows an increased Cho/NAA ratio within the mass (green arrows in f). No parenchymal extension or edema is seen

MRI showed a lobulated mass with intense homogeneous post-contrast enhancement (Figure 2). It was slightly heterogeneous and of predominantly intermediate signal intensity on both T1WI (similar to white matter) and T2WI (similar to grey matter). It did not demonstrate greater diffusion restriction than the brain parenchyma. Few dark foci of blooming were present on SWI corresponding to the calcifications seen on CT. MRS showed an increased choline/N-acetyl aspartate (Cho/NAA) ratio. The internal cerebral veins were stretched and displaced laterally by the mass. No parenchymal extension of the mass or surrounding edema was noted. A differential diagnosis of an intraventricular neoplasm such as meningioma, ependymoma, and choroid plexus papilloma was suggested.

The patient underwent endoscopic resection of the intraventricular tumor. The tumor was firm and calcified and appeared to arise from the choroid plexus. Histopathology revealed a papillary lesion displaying delicate fibro-vascular cores covered by a single layer of fairly uniform cuboidal to low columnar epithelial cells with round to oval basally located monomorphic nuclei. No mitosis, necrosis, or anaplastic features were seen. Focal areas of calcification were noted within the papillae and stroma. Immunohistochemical studies showed tumor cells immuno-reactive with Vimentin, synaptophysin, S-100, EMA (weak and focal), GFAP (focal), and CK7 (focal); and negative with CK20, p53, and TTF-1. Ki-67 proliferative index was approximately 2%. A histopathological diagnosis of CPP was made.

**Figure 3:**
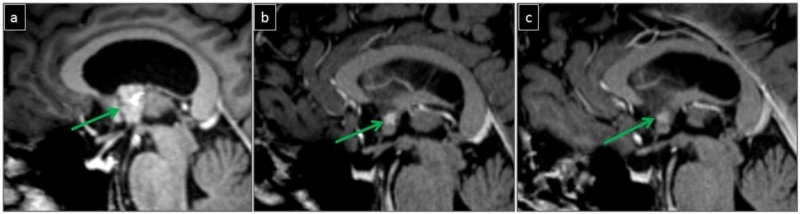
MRI brain (a-c). Sagittal T1-fat saturated post-contrast images from the preoperative scan showing the enhancing mass in the anterior third ventricle (green arrow in a). Initial post-operative scan after six weeks shows a tiny enhancing residual component (green arrow in b), which is stable in the follow-up scan after one year (green arrow in c)

Post-operative MRI performed six weeks after surgery showed a tiny residual enhancing tumor component, which was stable in size in a follow-up MRI performed one year later (Figure 3). One year after surgery, the patient complained of mild intermittent right-sided headache which was different from the headache he experienced prior to surgery.

## Discussion

The initial diagnosis of a colloid cyst in our case was mainly based on its location and features on CT. Colloid cysts are the commonest masses encountered in the third ventricle, usually located along the roof at the foramen of Monro, and are typically hyperdense on unenhanced CT. However, in view of the calcifications within the mass, alternative diagnoses should have been considered as calcifications are rare in colloid cysts [7]. Other lesions that can occur at this site and are more likely to contain calcifications include meningioma, choroid plexus tumor, ependymoma, and craniopharyngioma; and in particular, meningiomas and choroid plexus tumors are often hyperdense on CT [2-4].

The intense homogeneous enhancement of the mass on MRI excluded the diagnosis of a colloid cyst which would be non-enhancing or have only a thin peripheral rim of enhancement. There was no vascular communication of the mass to suggest an aneurysm. The enhancement pattern coupled with the elevated Cho/NAA ratio of the mass on MRS was indicative of a neoplasm. Purely intraventricular neoplasms within the third ventricle can be broadly categorized into neoplasms arising from the choroid plexus (including CPP, atypical CPP, and CPC) and neoplasms arising from other than the choroid plexus (such as ependymoma, meningioma, craniopharyngioma, chordoid glioma), metastases and lymphoma [4]. Of these, metastases, meningiomas, choroid plexus tumors, and chordoid gliomas often show avid post-contrast enhancement. 

Metastasis should always be considered in the differential of an avidly enhancing intraventricular mass lesion; however, this was less likely in our patient who had no known primary malignancy. Chordoid gliomas are rare neoplasms encountered in adults, they are usually hyperdense on unenhanced CT and show intense post-contrast enhancement; calcifications are rare [8]. Ependymomas typically demonstrate heterogeneous enhancement with cystic components. Craniopharyngiomas are typically sellar or suprasellar, along the floor of the third ventricle (rather than the roof), and are more heterogeneous in appearance with cystic changes. Other lesions that occur near the foramen of Monro and contain calcifications include subependymal giant cell tumors (in patients with tuberous sclerosis) and subependymomas (which usually only show minimal enhancement).

On the basis of imaging features, the most likely possible differential diagnosis was either a meningioma or a choroid plexus tumor. Intraventricular meningiomas commonly occur in patients aged 30-60 years; they are usually well-defined iso to hyperdense globular masses, with calcification (in over 50%) and show intense enhancement; they are iso to hypointense on T1W1 and iso to hyperintense on T2WI; they demonstrate increased blood volume on perfusion imaging [2,3]. Choroid plexus tumors are usually well-circumscribed, lobulated, iso to hyperdense, avidly enhancing, and may contain calcifications (in over 20%); they are iso to hypointense on T1W1 and of variable hyperintensity on T2WI; they show increased blood volume on perfusion imaging [2-3]. On MR spectroscopy, both meningiomas and CPTs show elevated choline; furthermore, increased lactate favors CPC over CPP [3,9,10]. In the case of a CPT, increased heterogeneity (necrosis) and parenchymal invasion favor a diagnosis of CPC. Seeding of the cerebrospinal fluid has been reported with both CPP and CPC (far more common with CPC); MRI of the spinal axis is therefore recommended [2,11,12].

Our patient underwent endoscopic resection of his intraventricular tumor, and histopathology revealed a CPP. Choroid plexus tumors are primarily treated with surgical resection, which may be followed by adjuvant therapy (chemotherapy and/or radiotherapy) in cases of CPC [12,13]. Gross total resection is associated with higher survival rates and lower rates of recurrence [12]. Relapse is a poor prognostic factor in cases of CPC, but not in CPP [13]. The five-year survival rate for CPP is over 95%, whereas in CPC it is less than 45% [3].

## Conclusions

Although a well-circumscribed hyperdense mass in the third ventricle near the foramen of Monro on an unenhanced CT scan is most likely to be a colloid cyst, further assessment by contrast-enhanced CT and/or MRI should be performed to exclude alternative diagnoses. Third ventricle neoplasms are uncommon and preoperative differentiation can be challenging due to overlap in imaging features. However, awareness of the imaging features of different neoplasms on CT and MRI can help narrow the differential diagnosis.
